# Immunocytochemical characterisation of cultures of human bladder mucosal cells

**DOI:** 10.1186/1471-2490-11-5

**Published:** 2011-04-18

**Authors:** Jacqueline R Woodman, Kylie J Mansfield, Vittoria A Lazzaro, William Lynch, Elizabeth Burcher, Kate H Moore

**Affiliations:** 1 Detrusor Muscle Laboratory, The St George Hospital, University of New South Wales, Sydney, NSW 2052, Australia; 2 Graduate School of Medicine, University of Wollongong, Wollongong NSW 2522, Australia; 3 Department of Pharmacology, School of Medical Sciences, University of New South Wales, Sydney, NSW 2052, Australia; 4 University Hospital Coventry and Warwickshire (UHCW), Coventry, CV2 2DX, UK; 5 Miltenyi Biotech, Sydney, NSW, Australia

**Keywords:** urothelial cells, myofibroblasts, immunocytochemistry, human

## Abstract

**Background:**

The functional role of the bladder urothelium has been the focus of much recent research. The bladder mucosa contains two significant cell types: urothelial cells that line the bladder lumen and suburothelial interstitial cells or myofibroblasts. The aims of this study were to culture these cell populations from human bladder biopsies and to perform immunocytochemical characterisation.

**Methods:**

Primary cell cultures were established from human bladder biopsies (n = 10). Individual populations of urothelial and myofibroblast-like cells were isolated using magnetic activated cell separation (MACS). Cells were slow growing, needing 3 to 5 weeks to attain confluence.

**Results:**

Cytokeratin 20 positive cells (umbrella cells) were isolated at primary culture and also from patients' bladder washings but these did not proliferate. In primary culture, proliferating cells demonstrated positive immunocytochemical staining to cytokeratin markers (AE1/AE3 and A0575) as well fibroblasts (5B5) and smooth muscle (αSMA) markers. An unexpected finding was that populations of presumptive urothelial and myofibroblast-like cells, isolated using the MACS beads, stained for similar markers. In contrast, staining for cytokeratins and fibroblast or smooth muscle markers was not co-localised in full thickness bladder sections.

**Conclusions:**

Our results suggest that, in culture, bladder mucosal cells may undergo differentiation into a myoepithelial cell phenotype indicating that urothelial cells have the capacity to respond to environmental changes. This may be important pathologically but also suggests that studies of the physiological function of these cells in culture may not give a reliable indicator of human physiology.

## Background

The urinary bladder is lined by a transitional epithelial layer, the urothelium. For many years, the urothelium was thought to be merely a passive barrier, preventing egress of solutes into the bladder wall. In the last decade, it has been shown that the urothelial layer expresses muscarinic [1-3] vanilloid [4] and purinergic receptors [5]. Recent evidence suggests that mediators such as nitric oxide [6], adenosine triphosphate [7] and acetylcholine [8,9] are also released from this urothelial layer. Changes in the urothelium have been associated with several disorders of the lower urinary tract in man [4,10-14]. Hence clinicians and scientists have developed increasing interest in the morphology and function of this complex layer. A lack of access to normal human urothelium has hampered investigations into the pathophysiological roles of the urinary bladder and the changes associated with disease in humans. Most cell culture studies have been performed using animal bladders [15] where cells can be isolated from relatively large amounts of bladder tissue.

Small amounts of human bladder tissue are obtainable from biopsy specimens; however this imposes limits on cell culture research. Thus human studies tend to focus upon full thickness bladder samples obtained from open bladder surgery [16]. These studies are unable to provide information about the functional roles of the individual components of the bladder mucosa. In addition, full thickness bladder specimens are not available from patients with bladder dysfunctions such as incontinence, because they do not generally have open bladder surgery.

The bladder mucosa is made up of the urothelium with an underlying lamina propria containing myofibroblasts, blood vessels and nerves. The urothelium is a multilayered transitional epithelium which displays a regular architecture. The basal cells, situated on the basement membrane, differentiate through a number of intermediate cells to the highly differentiated superficial or umbrella cells [17]. Normal human urothelial cells are able to proliferate and regenerate in culture but do not express markers of urothelium differentiation [18]. When grown in culture, human urothelial cells form a partially differentiated urothelium [19] indicating that in culture urothelial cells retain the potential to undergo cytodifferentiation [20].

Beneath the urothelial cells is a layer of interstitial cells, which have the functional and histological characteristics of myofibroblasts [21] and exhibit close connections to suburothelial nerves [22,23]. Thus researchers have become increasingly interested in the functional roles of both the urothelium and the adjacent myofibroblasts.

The aims of this study were to develop a reliable culture method for use in human bladder biopsy specimens. Specifically, we characterised the immunocytochemical features of the urothelial and myofibroblast cell layers during cell culture and subsequent passage. To separate different cell types, we used a cell enrichment technique known as magnetic activated cell separation of human cells (MACS). This technique enables isolation of functionally active cells, the isolation of rare cells and the recovery of intact cells with minimal stress during sorting [24]. To strengthen the characterisation of these cells, we undertook immunocytochemical analysis of full thickness bladder samples, taken at open surgical procedures.

## Methods

### Patients and specimens

Human bladder biopsies were collected from 10 patients undergoing cystoscopic examination for asymptomatic haematuria or previously treated low-grade bladder cancer. Subsequent histopathology revealed that the biopsies were not malignant. Macroscopically normal full thickness segments of urinary bladder wall from 3 patients (65 to 71 years) were obtained at open operation. Bladder washings were collected at cystometry from 9 female patients with stress urinary incontinence. Informed consent for collection of the above specimens was obtained, in accordance with the local hospital human ethics committee approval.

### Primary culture and preparation of cell suspensions from biopsy specimens

Biopsies were obtained at cystoscopy from a site 2 cm cephalic and 2 cm lateral to the left ureteric orifice. Bladder biopsies measuring approximately 1.5 - 6 mm^3 ^(weight 10 - 70 mg) collected into sterile saline using strict aseptic procedures, or into culture medium (RPMI 1640) supplemented with penicillin (100 units/ml), streptomycin (100 μg/ml) and fungazone (0.25 μg/ml).

Immediately after collection, the mucosa was dissected away from any underlying detrusor smooth muscle, which was discarded together with blood vessels and connective tissue. The dissected mucosa was washed with media (as above, three times) and then dissociated by digestion with trypsin-EDTA (0.25 g trypsin (1:250) and 0.38 g EDTA) at 37°C in 95% air, 5%CO_2 _for 5 to 10 min.

The trypsin was inactivated with an equal volume of growth medium at 4°C, and the digested tissue passed through a 100 μm nylon cell strainer and the cell suspension centrifuged at 428 g for 10 min at 4°C. The cell pellet was re-suspended in growth medium (RPMI1640 supplemented with penicillin (100 units/ml), streptomycin (100 μg/ml) and fungazone (0.25 μg/ml) glutamine 2 mM, HEPES Buffer 25 mM and 10% heat deactivated foetal bovine serum). Each biopsy yielded approximately 2 × 10^5 ^cells. Cells were plated in 25 cm^2 ^flasks (Becton Dickinson) or 96 well plates and maintained in a minimum volume of culture medium for several hours at 37°C in 95% air, 5% CO_2 _to encourage cell attachment. Thereafter, further culture medium was added to adequately cover the cells and changed every three days until approximately 80% confluence was reached.

### Isolation of cell populations using MACS Microbeads

Individual urothelial and fibroblast cell populations were isolated using the MACS anti-fibroblast microbeads at the first or second passage [25]. The enrichment protocol was carried out as described by the manufacturers.

Cells were passaged using trypsin-EDTA (5 min at 37°C) and the cell suspension centrifuged at 100 g for 10 min at room temperature. The cell pellet (~10^7 ^cells) was resuspended in buffer (phosphate buffered saline (pH7.2) supplemented with 0.5% bovine serum albumin, 80 μl) and microbeads (20 μl). The resulting suspension was incubated for 30 min at room temperature then washed in buffer (1 ml) and centrifuged at 100 g for 10 min. The supernatant was discarded and the cell pellet resuspended in buffer (1 ml buffer), filtered through a 30 μm nylon mesh and loaded onto the magnetic separation (MS) column which was placed in a magnetic field. Unlabeled cells were washed though the column (3× with buffer). The column was then removed from the magnetic field and the column washed with buffer to elute the labelled or bound cells. To achieve a higher purity, the labelled cells were loaded onto a new, freshly prepared column and the process repeated. At the completion of each cell enrichment procedure, two-cell suspensions were obtained: the eluted cells (urothelium) and the bound cells (myofibroblast-like cells). The cell suspensions were diluted in culture medium and centrifuged 190 g for 5 min, supernatant discarded, cell pellet resuspended in culture medium. Each cell suspension was cultured into 12.5 cm^2 ^flasks and 96 well plates using culture media and incubation conditions as described above.

### Characterisation of superficial (umbrella) cells from bladder washings

Bladder washing fluid was collected into a flask primed with 100 ml of culture media (as above). The collected solution was passed through a 100 μm mesh and decanted into 50 ml centrifuge tubes before being centrifuged at 428 g for 10 min at 4°C. The supernatant was discarded and the cell pellet resuspended in culture medium. This was centrifuged at 122 g for 10 min at 4°C and the cell pellet resuspended in growth media and cultured (as above). Trypan blue was used to examine cell viability prior to culture.

### Identification of cell populations in culture, using immunocytochemistry

Selective primary antibodies (Table 1) were employed to identify the cell types present in individual cultures, using immunocytochemistry. Positive and negative controls were used to confirm specificity for αSMA and cytokeratin markers.

**Table 1:** Selectivity of the individual primary antibodies used to characterise cell populations

Primary Antibody	Dilution used	Antibody selectivity
AE1/AE3	1:1000	Cytokeratins present in epithelial cells

A0575	1:500-1:1000	Cytokeratins present in epithelial cells

Cytokeratin 20 (CK20)	1:200	Superficial umbrella cells

α-smooth muscle actin (αSMA)	1:200	Smooth muscle, myofibroblasts and myoepithelial cells, not fibroblasts

5B5	1:100	Fibroblast and myoepithelial cells

Culture media was aspirated from plated cells which were then rinsed three times with wash buffer (50 mM Tris buffered saline with 0.05% Tween 80). Cells were fixed with absolute ethanol for 5 min then allowed to air dry for 30 min. Cells were permeabilised with wash buffer for 30 min at room temperature. Primary antibody (Table 1) was added to the cells and incubated for 1 h at 37°C followed by three rinses with wash buffer. Envision AP polymer conjugate was added to the cells and incubated for 30 min at 37°C followed by three rinses with wash buffer before Fast Red chromogen solution was added. The cells were incubated initially for 5 min at room temperature, before a further application of chromogen and a further 10 min incubation at room temperature. The chromogen solution was aspirated and the cells rinsed with distilled water.

### Histology of full thickness bladder specimens

Macroscopically normal full thickness segments of urinary bladder wall were collected into carbogenated Krebs-Henseleit solution (composition in mM: NaCl 118, KCl 4.7, NaHCO_3 _25, KH_2_PO_4 _1.2, MgSO_4 _1.2, CaCl_2 _2.5 and D-glucose 11.7), and transported immediately to the laboratory. Segments were dissected and pinned stretched onto cork boards and fixed in Zamboni's fixative. Segments were washed three times in DMSO followed by three washes in 0.1 M phosphate buffered saline before being placed in 30% sucrose in 0.1 M phosphate buffered saline. Segments were submerged in Tissuetek^® ^and frozen slowly in liquid nitrogen then stored at -80°C. For immunohistochemistry, 5-7 μm thick sections were mounted onto Super Frost Plus Slides (Menzel) and air-dried. Each slide was double labelled with the same primary antibodies used for cell cultures studies, using Dako Envision G/2 double labelling kit.

### Analysis

Positive immunocytochemical signals were indicated by the presence of distinct red coloured cytoplasmic precipitate at the antigen site. The immunoreactive cells were assessed visually by phase contrast microscopy. The positive staining was expressed as the % red cytoplasmic staining of the total area of growth cultures. The results are expressed as follows: 0 (no staining detected), + some staining (1-10% of total area of growth with positive staining), ++ (>10 - 40% of total area of growth with positive staining), +++ (>40 - 70% of total area of growth with positive staining) and ++++ predominant staining (>70 - 100% of total area of growth with positive staining).

### Materials

Unless otherwise specified, all tissue culture materials were purchased from Invitrogen and plastic ware from Becton Dickson. MACS Microbeads were purchased from Miltenyi Biotec. All antibodies, including negative controls, were from DakoCytomation and the visualisation system was the Universal DakoCytomation EnVision System-Alkaline Phosphatase AP with Fast Red Substrate-Chromogen and Envision Labelled Polymer, Alkaline Phosphatase.

## Results

### Expression of markers in primary mucosal cell cultures from biopsy specimens

Single cell suspensions were produced by trypsin digestion of mucosal cells, with the isolated cells adhering to the surface of the culture plate within several hours of plating. The total cell yield varied considerably, depending on the size of the biopsy. Cells were slow to grow, with 80% confluence obtained after 3 to 5 weeks in culture.

**Figure 1:**
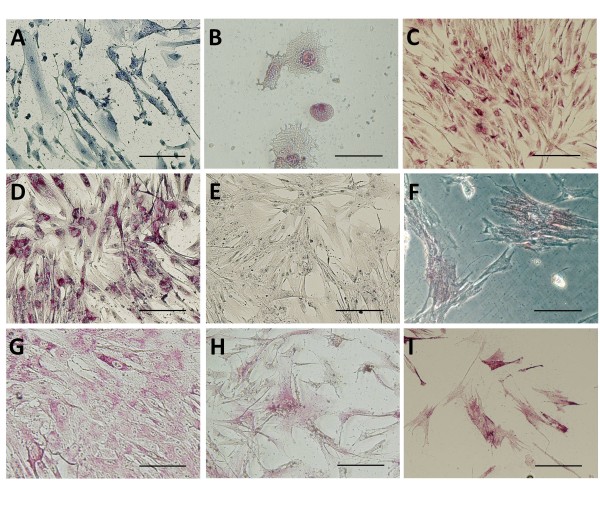
**Immunohistochemistry of cultured bladder mucosal cells**. At primary culture (A, B, C) cells were counter stained to demonstrate morphology (A). A small number of cells were large with a spreading morphology, these stained for CK20 (B). Other cells were more elongated and branching and stained for AO575 (C). Cells were also stained after treatment with MACS beads. Cells that bound to the beads (D, E, F, myofibroblast-like cells) were stained for 5B5 (D), AE1/AE3 (E) and αSMA (F). Cells that eluted from the beads (G, H, I, urothelial cells) were stained for 5B5 (G), A0575 (H) and αSMA (I). (Bar = 100 μm)

In preliminary immunocytochemical studies (n = 2, Figure 1), cells in primary culture were found to display diverse morphologies (Figure 1A). Large spreading umbrella-like cells were isolated early in culture, but these cells did not proliferate. Immunostaining of cultures containing these large spreading cells demonstrated that they expressed CK20 (Figure 1B).

Other mucosal cell types proliferated strongly in primary culture and these were positive for other cytokeratins (AE1/AE3 and A0575 (Figure 1C). These cell populations developed a fine network of extensions (Figure 1C) that appeared to connect one cell to another. It was notable that these cultures were also positive for the smooth muscle marker αSMA and the fibroblast marker 5B5 (Table 2), markers not usually associated with urothelial cells.

**Table 2:** Characterisation of cell populations present in human bladder mucosa cultures

	A0575	AE1/AE3	CK20	αSMA	5B5
Primary culture	++ (n = 2)	++ (n = 2)	+++ (n = 2)	++ (n = 2)	++ (n = 2)

Anti-fibroblast eluted	++++ (n = 7)	++ (n = 5)	0 (n = 2)	++ (n = 7)	+++ (n = 4)

Anti-fibroblast bound	++++ (n = 7)	0 (n = 5)	0 (n = 2)	+++ (n = 5)	+++ (n = 7)

The results are expressed as follows: 0 (no staining detected), + some staining (1-10% of total area of growth with positive staining), ++ (>10 - 40% of total area of growth with positive staining), +++ (>40 - 70% of total area of growth with positive staining) and ++++ predominant staining (>70 - 100% of total area of growth with positive staining).

### Isolation of individual cell populations using anti-fibroblast MACS microbeads

Anti-fibroblast MACS beads were used to separate the myofibroblast-like cells from the urothelial cells within the primary cultures. The resulting cultures were either derived from cells that were eluted (anti-fibroblast negative, urothelial cells) or bound (anti-fibroblast positive, myofibroblast-like cells).

The anti-fibroblast positive cells that were bound to the beads displayed immunoreactivity for 5B5 (Figure 1D) as well as for the cytokeratins (AE1/AE3, Figure 1E) and αSMA (Figure 1F). Cells that were anti-fibroblast negative and were eluted from the columns displayed immunoreactivity to the cytokeratin markers (A0575, Figure 1H); these cultures also showed immunoreactivity for 5B5 (Figure 1G) and αSMA (Figure 1I).

**Figure 2:**
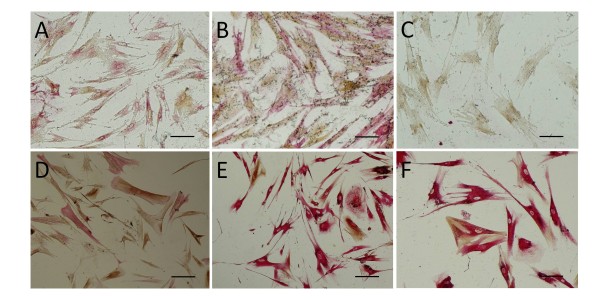
**Dual labeling of MACS treated cell populations**. Cells that bound to the MACS anti-fibroblast beads (myofibroblast-like cells, A, B, C) or that were eluted from the MACS beads (urothelial cells, D, E, F) were stained with (A, D) A0575 (brown) and 5B5 (Red) (B, E) with A0575 (brown) and αSMA (Red) and (C, F) αSMA (brown) and 5B5 (Red). (Bar = 100 μm)

Because MACS treated cultures showed similarities in immunocytochemical staining, dual labeling was performed on both bound and eluted populations (Table 2). The anti-fibroblast positive cells (myofibroblast-like cells) that were bound to the beads demonstrated dual labelling for cytokeratin markers together with 5B5 and αSMA (Figure 2A, B, C). Similarly, cells that were anti-fibroblast negative (urothelial cells) that were eluted from the columns also displayed the same pattern of dual labelling for cytokeratins together with 5B5 and αSMA (Figure 2D, E, F).

### Characterisation of superficial umbrella cells from bladder washings

**Figure 3:**
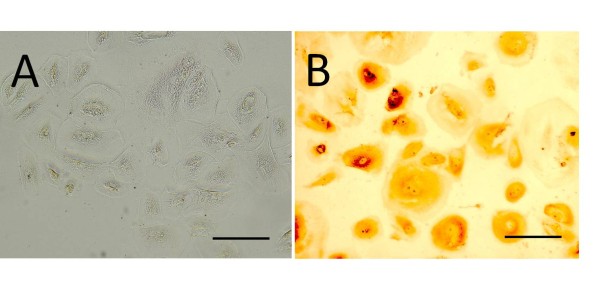
**Bladder superficial urothelial cells isolated from bladder washings**. Phase contrast microscopy demonstrated large spreading cells (A) which were positive for the umbrella cell marker CK20 (B). (Bar = 50 μm)

The cells isolated from the bladder washings were large spreading cells (Figure 3A) that were positive for A0575 and CK20, a marker indicative of fully differentiated superficial umbrella cells(Figure 3B). At the time of isolation the isolated umbrella cells were able to exclude trypan blue indicating cell viability. However, similar to the CK20-positive cells isolated from bladder biopsy specimens, these cells did not proliferate in culture.

### Immunohistochemical staining of full thickness bladder segments

**Figure 4:**
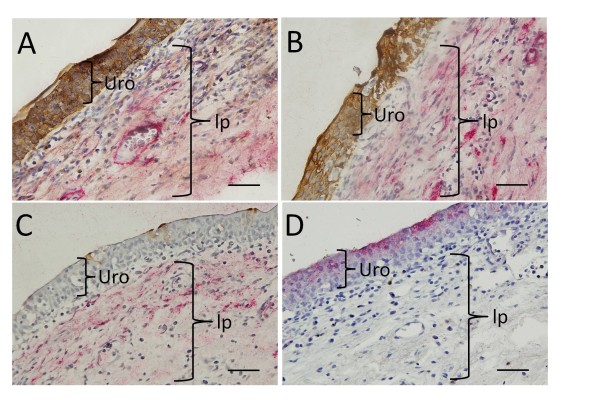
**Dual labelling of urothelim (Uro) and lamina propria (lp) of human bladder**. Full thickness segments of human bladder were stained with (A) the cytokeratin marker A0575 (brown) and αSMA (red) or (B) AE1/AE3 (brown) and αSMA (red). (C) with CK20 (brown) and αSMA (red). D. Full thickness segments were stained with 5B5 (red). Sections were counterstained with haematoxylin. (Bar = 50 μm)

To confirm the localization of the antibodies used in this study immunohistochemical staining was also performed in full thickness bladder segments (Figure 4). The urothelium demonstrated strong immunoreactivity for the cytokeratin markers (A0575, Figure 4A and AE1/AE3, Figure 4B). Although many of the superficial umbrella cells were lost during processing, the remaining superficial cells demonstrated immunoreactivity to CK20 (Figure 4C). None of the urothelial cells stained positively for αSMA (Figure 4A, B and 4C).

However, it was notable that the upper quarter of the urothelial layer also demonstrated immunoreactivity for the fibroblast marker 5B5 (Figure 4D). Furthermore, there was a discontinuous layer just below the basal urothelial cells that showed strong immunoreactivity to αSMA (Figure 4B and 4C). In the lamina propria, the blood vessels were easily identified by the positive αSMA staining (Figure 4A).

## Discussion

In this study we have shown that small human bladder biopsy specimens taken from patients with non-cancerous conditions can be cultured. The growth rate of the cultures established from these bladder biopsies was relatively slow, taking 3 to 5 weeks to reach confluence. Bladder urothelial cells are known to be slow growing with a previous study isolating urothelial cells from large bladder cystectomy samples reported confluence only being reached in 20% of cases even after 2 to 3 weeks in culture [26]. In this study the slow growth rate is likely related to the small number of cells (approximately 2 × 10^5 ^cells isolated from each biopsy) isolated from the biopsy tissue samples [27]. In addition, our cultures were established from bladder biopsies taken from adults (age range 19 to 85) which influences the rate of urothelial cell growth in culture [26-28].

As expected, urothelial cells cultured from biopsy samples showed positive immunoreactivity for cytokeratin markers (CK20, AE1/AE3 and A0575). However, an unexpected finding was that some cells in the culture also demonstrated immunoreactivity for markers of smooth muscle (αSMA). This was anomalous as in full thickness bladder segments, αSMA staining was not present in the urothelial layer, rather positive staining for αSMA was seen in a sub-urothelial layer, which was consistent with a myofibroblast layer previously shown in urinary bladder from the pig [21]. However, this myofibroblast cell layer does not stain for cytokeratin markers. This pattern of immunocytotochemical staining of cells in culture therefore indicated that either the urothelial cells have the potential to develop morphological features of cells that possess contractile properties (i.e. myoepithelial cells) or that mixed populations of both urothelial cells and myofibroblasts from lamina propia were present in the cultures. Mixed populations of cobblestone and spindle shaped cells have been previously described in cultures from the urinary tract [28].

Anti-fibroblast MACS microbeads were used to isolate individual populations of urothelial and myofibroblast-like cells. We expected to obtain two individual populations. Firstly, a population of myofibroblast-like cells, which were anti-fibroblast positive cells that bound to the MACS beads and secondly, a population of urothelial cells, which were anti-fibroblast negative cells that were eluted from the beads. However, in these experiments both the presumptive urothelial and myofibroblast-like cells were found to stain for the same markers (AO575, 5B5 and αSMA). This could be explained if the cells isolated had the capacity to express characteristics of epithelial (AO575), fibroblast (5B5) and smooth muscle (αSMA) cells during culture.

It is known that epithelial cells such as the urothelium have the capacity to undergo transformation. Epithelial-mesenchymal-myofibroblast transformation is usually induced by growth factors e.g. TGF-β [29-33]. This transformation is a key feature in conditions such as organ fibrosis and wound healing [29]. These transformed epithelial cells will then characteristically express αSMA [30-32]. Such transformations are commonly associated with injury of cell-cell junctions [31,33]. Cross and associates [20] suggested that transformation of urothelial cells could be influenced by the culture conditions and demonstrated the reversibility of differentiation between squamous and transitional cell phenotype. Interestingly, previous studies have reported that prolonged culture of urothelial cells isolated from bladder [26] and ureter [34] is associated with the co-expression of cytokeratins and vimentin. Vimentin is a marker commonly used for myofibroblasts [35-38]. It could be suggested that the changes in urothelial cells in culture may occur due to the "damage" the cells perceive when they are isolated for culture. Alternatively, this may represent naturally occurring process of transdifferentiation *in vivo*.

The expression of αSMA in cultured urothelial cells implies that urothelial cells have the ability to acquire contractile components given the appropriate stimulus. One stimulus for acquisition of contractile components could be cell damage in response to inflammation or another insult. One *in vivo *model of urothelial cell damage is treatment with cyclophosphamide, an irritant that is excreted in the urine. Cyclophosphamide use has been associated with the identification of actin filaments localised to rat urothelial cells [39]. Similarly, activation of inflammatory mast cells has been associated with transformation of fibroblasts, as indicated by expression of αSMA [40]. Together, these findings could be important to our understanding of bladder pathology, since inflammation is currently being considered as having a role in the aetiology of bladder conditions such as interstitial cystitis [41] and the overactive bladder [42,43].

In all cultures the isolated cells expressed cytokeratins, as indicated by positive immunocytochemical staining with the markers AE1/AE3 and A0575. Of interest is the difference in the pattern of cytokeratin staining in the anti-fibroblast positive cells. These cells stained positively for A0575 but not for AE1/AE3. The difference in this result could be related to differences in the cytokeratins labelled by the different antibodies. The AE1/AE3 antibody identifies the majority of human cytokeratins present in epithelial cells but not CK12, CK17 or CK18. In contrast the A0575 antibody is a cocktail of low and high molecular weight cytokeratins. The differences in the pattern of cytokeratin staining in the anti-fibroblast positive cells could also relate to the state of differentiation of these cells as cytokeratin expression in the urothelium alters with differentiation of urothelial cells in culture [26,27] and with the and position within the layers of cells making up the urothelium *in vivo *[44].

It is known that normal urothelium is characterised by terminally differentiated superficial cells, which express cytokeratin 20 [44-46]. While our early primary cultures derived from bladder biopsies contained some cells that were positive for CK20, this marker was soon lost in culture. To confirm that these cells were in fact terminally differentiated, superficial umbrella cells, we isolated cells from bladder washings obtained during cystometric testing (Figure 3). During cystometric testing the bladder is filled with saline in the presence of a catheter which may dislodge some of the superficial umbrella cells. The cells isolated from bladder washings showed similar growth and staining patterns to the CK20 positive cells isolated from the bladder biopsy specimens. Our finding that CK20 staining is lost with culture is in keeping with previous work showing that in culture, human urothelium does not undergo complete terminal differentiation and does not express cytokeratin 20 [19,47,48].

## Conclusions

In conclusion, it appears that primary cultures of urothelial and myofibroblast-like cells from human bladder biopsies are quite feasible, but the cells are slow growing, needing at least 3 weeks to attain confluence. Our results suggest that urothelial cells in culture alter their characteristics, so that studies of physiological function in these cells may not give a reliable indicator of human physiology *in vivo*. However, these results demonstrating transformation of urothelial cell into a contractile phenotype may have important implications for understanding of bladder dysfunction.

## Abbreviations

αSMA: α smooth muscle actin; EDTA: ethylenediaminetetraacetic acid; HEPES: 4-(2-hydroxyethyl)-1-piperazineethanesulfonic acid; MACS: magnetic activated cell separation

## Competing interests

The authors declare that they have no competing interests.

## Authors' contributions

JRW and KJM conceived of the study, and participated in its design and coordination and helped to draft the manuscript. VAL carried out the cell culture and immunocytochemistry. WL and KHM provided human bladder specimens. KHM and EB participated in the design of the study and helped to draft the manuscript. All authors read and approved the final manuscript.

## Pre-publication history

The pre-publication history for this paper can be accessed here:

http://www.biomedcentral.com/1471-2490/11/5/prepub
